# Impact of short and long exposure to cafeteria diet on food intake and white adipose tissue lipolysis mediated by glucagon-like peptide 1 receptor

**DOI:** 10.3389/fendo.2023.1164047

**Published:** 2023-05-24

**Authors:** Pamela Mattar, Cristian Jaque, Jennifer A. Teske, Eugenia Morselli, Bredford Kerr, Víctor Cortés, Rene Baudrand, Claudio E. Perez-Leighton

**Affiliations:** ^1^ Facultad de Ciencias Biológicas, Pontificia Universidad Católica de Chile, Santiago, Chile; ^2^ Department of Physiology, School of Nutritional Sciences and Wellness, Graduate Interdisciplinary Programs in Physiological Sciences and Neuroscience, University of Arizona, Tucson, AZ, United States; ^3^ Department of Food Science and Nutrition at the University of Minnesota, Saint Paul, MN, United States; ^4^ Department of Basic Sciences, Faculty of Medicine and Sciences, Universidad San Sebastián, Santiago, Chile; ^5^ Centro de Biología Celular y Biomedicina-CEBICEM, Facultad de Medicina y Ciencia, Universidad San Sebastián, Santiago, Chile; ^6^ Department of Nutrition, Diabetes, and Metabolism, Faculty of Medicine, Pontificia Universidad Católica de Chile, Santiago, Chile; ^7^ Department of Endocrinology, Faculty of Medicine, Pontificia Universidad Catolica de Chile, Santiago, Chile; ^8^ Centro Traslacional de Endocrinologia UC CETREN, Pontificia Universidad Catolica de Chile, Santiago, Chile

**Keywords:** obesity, glucagon-like peptide 1 (GLP-1), cafeteria diet, lipolysis, white adipose tissue

## Abstract

**Introduction:**

The modern food environment facilitates excessive calorie intake, a major driver of obesity. Glucagon-like peptide 1 (GLP1) is a neuroendocrine peptide that has been the basis for developing new pharmacotherapies against obesity. The GLP1 receptor (GLP1R) is expressed in central and peripheral tissues, and activation of GLP1R reduces food intake, increases the expression of thermogenic proteins in brown adipose tissue (BAT), and enhances lipolysis in white adipose tissue (WAT). Obesity decreases the efficiency of GLP1R agonists in reducing food intake and body weight. Still, whether palatable food intake before or during the early development of obesity reduces the effects of GLP1R agonists on food intake and adipose tissue metabolism remains undetermined. Further, whether GLP1R expressed in WAT contributes to these effects is unclear.

**Methods:**

Food intake, expression of thermogenic BAT proteins, and WAT lipolysis were measured after central or peripheral administration of Exendin-4 (EX4), a GLP1R agonist, to mice under intermittent-short exposure to CAF diet (3 h/d for 8 days) or a longer-continuous exposure to CAF diet (24 h/d for 15 days). *Ex-vivo* lipolysis was measured after EX4 exposure to WAT samples from mice fed CAF or control diet for 12 weeks. .

**Results:**

During intermittent-short exposure to CAF diet (3 h/d for 8 days), third ventricle injection (ICV) and intra-peritoneal administration of EX4 reduced palatable food intake. Yet, during a longer-continuous exposure to CAF diet (24 h/d for 15 days), only ICV EX4 administration reduced food intake and body weight. However, this exposure to CAF diet blocked the increase in uncoupling protein 1 (UCP1) caused by ICV EX4 administration in mice fed control diet. Finally, GLP1R expression in WAT was minimal, and EX4 failed to increase lipolysis *ex-vivo* in WAT tissue samples from mice fed CAF or control diet for 12 weeks. .

**Discussion:**

Exposure to a CAF diet during the early stages of obesity reduces the effects of peripheral and central GLP1R agonists, and WAT does not express a functional GLP1 receptor. These data support that exposure to the obesogenic food environment, without the development or manifestation of obesity, can alter the response to GLP1R agonists. .

## 1 Introduction

The modern food environment is obesogenic. The easy access to various palatable foods rich in fat and carbohydrates results in excess calorie intake, increasing the risk of obesity and related diseases (1). This excessive calorie intake is driven by food intake despite hunger or satiety, a behavior called hedonic intake (2, 3). Further, repeated palatable food intake alters the mechanisms regulating food intake, thereby increasing hedonic intake and body weight gain (4). In rodents, the obesogenic environment is usually modeled by exposing rodents to diets enriched in a single macronutrient (i.e., fat or sucrose) (5). However, these diets do not model the hedonic intake driven by easy access to various palatable foods observed in the human obesogenic environment. The cafeteria (CAF) diet overcomes this limitation by using a rotating schedule of highly palatable human snacks and free access to standard rodent chow (6). The CAF diet causes hedonic intake and, over time, severe obesity (7, 8). Thus, the CAF diet is a valuable model to study not only diet-induced obesity but also the influence of the obesogenic environment on the control of feeding behavior and metabolism.

Glucagon-like peptide 1 (GLP1) is an anorexigenic neuroendocrine peptide that has been the basis for developing pharmacotherapies against obesity (9). Endogenous GLP1 is released post-prandially by neuroendocrine L cells of the small and large intestines and by neurons in the nucleus of the solitary tract that express the pre-proglucagon peptide (PPG-NTS neurons) (10–12). In rodents, activating the GLP1 receptor (GLP1R) in peripheral tissues (i.e., vagal afferents, enteric neurons) or different brain regions can reduce food intake (12). Still, the reduction in weight loss and weight loss caused by activation of peripheral GLP1R do not require activation of PPG-NTS neurons (13, 14). In rodents, peripheral administration of exendin-4 (EX4), a long-lasting GLP1R agonist that crosses the blood-brain barrier, activates GLP1R in several hypothalamic nuclei (15), including the paraventricular nucleus (PVN). In this brain region, pharmacological activation of GLP1R or chemogenetic activation of GLP1R-expressing neurons reduces the intake of standard rodent food and decreases operant responding for sucrose (16–19). However, whether activation of central GLP1R, including in the PVN, can regulate hedonic intake in an obesogenic environment similar to the human food environment remains unclear.

In rodents, diet-induced obesity or the availability of palatable food reduces the anorectic effect of GLP1R activation by peripheral EX4 administration (20, 21). However, whether the same results would be observed after activating central GLP1R by the intracerebroventricular administration of EX4 is unclear. Also, whether this apparent resistance to EX4 administration results from exposure to palatable food or is the consequence of weight gain after exposure to a palatable diet remains unknown. This distinction is relevant as palatable food intake can elicit behavioral and metabolic effects independent of severe weight gain in rodents (22–24). Overall, whether exposure to an obesogenic environment before the onset or during early stages of obesity can reduce the anorectic effects of the central or peripheral administration of GLP1R agonists remains unclear.

Metabolic effects, in addition to reduced food intake, might explain the body weight loss caused by GLP1R activation. Central and peripheral administration of EX4 enhance white adipose tissue (WAT) lipolysis (25, 26), increase plasma free fatty acid (FFA) and triglyceride (TG) clearance, and promotes mitochondrial fatty acid oxidation in brown adipose tissue (BAT), WAT, and muscle (25, 27–30). Concordantly, central and peripheral administration of GLP1R agonists increases the expression of uncoupling protein 1 (UCP1) and other proteins involved in energy metabolism (i.e., Peroxisome proliferator-activated receptor gamma coactivator 1-alpha or carnitine palmitoyltransferase I) in WAT and BAT, leading to increased thermogenesis (27, 28, 31). Yet, two questions need to be answered regarding the effects of GLP1R agonists on WAT. First, it remains controversial whether exposure to palatable foods before the onset of obesity can decrease the impact of central and peripheral EX4 administration on BAT and WAT, or if this effect is observed only in obese animals (25–27). Second, it is unclear whether the effects of GLP1R agonists on WAT metabolism are mediated by direct activation of GLP1R in WAT, as the expression of a functional GLP1R in this tissue remains controversial (32, 33).

We previously showed that obesity caused by long-term and continuous access to a CAF diet (24 h/d for 10 weeks) reduced the anorectic effects of peripheral EX4 administration (21). Here we aimed to understand how exposure to an obesogenic environment modeled by CAF diet could alter the impact of EX4 on food intake, weight loss, and WAT metabolism. First, we examined whether different schedules of access during early exposure to a CAF diet before the onset of obesity reduced the anorectic effects of GLP1R activation by ICV and intraperitoneal (IP) EX4 administration. Second, we examined whether EX4 increased WAT lipolysis by acting in this tissue, and if this effect was also reduced by long-term exposure to a CAF diet.

## 2 Methods

### 2.1 Animals

All experimental protocols were approved by the Institutional Animal Care and Use Committee at Pontificia Universidad Catolica de Chile. Male C57BL/6J mice (originally obtained from Jackson Laboratories and bred at Pontificia Universidad Católica de Chile, 8-10-week-old at the beginning of experiments) were used in all experiments. Mice were maintained on a 12:12 h light:dark cycle in a temperature-controlled room (20-24°C) and had free access to standard rodent food (i.e., chow) and water, except where noted. Mice were grouped or singly housed in clear solid-bottom cages with paper bedding (2:1 mixture of sterilized shredded filter paper and paper towels) supplemented with environment-enriching materials. Mice were euthanized by isoflurane (Baxter) overdose at the end of each experiment.

### 2.2 Stereotaxic surgeries

Mice anesthetized with isoflurane (5% induction and 1-2% maintenance) were implanted with a single unilateral cannula (28 gauge, Plastics One) directed at the ventral third ventricle (ICV,-0.9 mm rostral, 0.0 mm lateral, 4.6 mm below skull surface) or PVN [-0.9 mm rostral, -0.2 mm lateral, 3.6 mm below skull surface (34)] using standard stereotaxic procedures (7, 35). Post-surgery care included ketoprofen (5 mg/kg, intraperitoneal injection) administered before, 24, and 48 h after surgery. After surgical recovery, mice were singly housed permanently and maintained without interventions for at least 7 days before beginning the experiments.

### 2.3 Drugs and injections

EX4 (#1933, Tocris Bioscience) and EX3 EX3-(9-39), #2081, Tocris Bioscience) were dissolved in sterile saline and stored at -20°C in single-use aliquots. Mice were acclimated to IP injections by receiving a daily IP injection (0.9% NaCl solution, 200 μL) for three consecutive days. Mice with ICV or PVN cannulae were acclimated to intra-cannula injections by receiving a single daily artificial cerebrospinal fluid (aCSF) injection, either ICV (0.5 μL) or intra-PVN (0.25 μL), respectively. All injections occurred within the last hour before lights off. After euthanasia, cannula placement was verified by histological methods (35). Data from mice with misplaced cannulae were excluded from the analyses.

### 2.4 *Ex-vivo* EX4 treatment of adipose tissue explants

Inguinal WAT (iWAT) and epididymal WAT (eWAT) depots were dissected into 50-100 mg pieces and incubated in DMEM culture media supplemented with 10% fetal bovine serum and antibiotics (penicillin-streptomycin, #03-031-1B, Biological Industries) at 37°C and 5% CO_2_ for 24 h (The medium was changed twice during this period). Next, WAT explants were treated with vehicle (saline), 100 μM isoproterenol (ISO), or 2.5 nM EX4 for 24 h, followed by KRH buffer (KRBH) plus free fatty acid bovine serum albumin (BSA) 4% for one h. Then, we quantified the release of glycerol into the KRBH medium with a glycerol colorimetric test (#F6428, Sigma). Glycerol release data were normalized by the protein concentration of the tissue explants.

### 2.5 Western blot

BAT, iWAT, and eWAT were homogenized in RIPA buffer (150 mmol/L NaCl, 10 mmol/L Tris base, 1% deoxycholic acid, 4.5 mM EDTA, and 1% Triton), supplemented with PhosStop phosphatase inhibitor cocktail (#4906845001, Roche) and COmplete^®^ protease inhibitor cocktail (#11836153001, Roche). The lysate was centrifuged (12,000 g for 15 min at 4°C), and the protein concentration was determined by a colorimetric assay (#23277, Pierce). The lysates were adjusted to 1-2 μg/μL protein in SDS-Page loading buffer (240 mmol/L Tris-HCl, pH 6.8, 8% SDS, 40% glycerol, and 20% 2-beta-mercaptoethanol) and heat-denatured at 100°C for 5 min. Twenty to thirty μg of protein were loaded on a polyacrylamide gel and electrophoresed at 80–120 V for 1.5 h. Depending on their size, the proteins were electrotransferred onto nitrocellulose 0.22 microns or polyvinylidene difluoride (PVDF) 0.45 microns membranes at 90 V for 1–1.5 h in a transfer buffer (24 mmol/L Tris, 194 mmol/L glycine, 20% methanol) and stained with Ponceau S for 5 minutes. Membranes were blocked with 5% non-fat milk or BSA in Tris-buffer saline (TBS) and incubated with primary antibodies (all hosted in Rabbit; pHSL (phospho-Hormone-Sensitive Lipase), #4139 Cell Signaling, 1:500; HSL (Hormone Sensitive Lipase), #4107 Cell Signaling 1:1000; UCP1, #293418 Santa Cruz Biotechnology, 1:100) overnight at 4°C. Immune complexes were detected using a peroxidase-conjugate secondary antibody (Goat anti-Rabbit #sc-2004, Santa Cruz Biotechnology) and the enzyme-substrate ECL (#20-500-500A and 20-500-500B, Biological Industries). Images were scanned and analyzed using ImageJ software. Protein bands were quantified by ImageJ software and normalized to total protein (for pHSL) or Ponceau S (for UCP1).

### 2.6 Isolation of total RNA, reverse transcription, and qPCR analysis

The medio basal hypothalamus, iWAT, and eWAT samples were lysed with TRIzol^®^ (#15596018 Invitrogen). Total RNA was isolated using PureLink™ RNA mini kit (#12183018A, Invitrogen). Reverse transcription was achieved using the High-Capacity cDNA Reverse Transcription kit (#4368813, Applied Biosystems). Quantitative polymerase chain reaction (qPCR) was performed in duplicates using the SYBR^®^FAST qPCR kit (#4385612, Applied Biosystems) with thermal conditions of 20-s preincubation at 95°C followed by 40 cycles at 95°C for 3 s and 60°C for 30 s. PCR primer sequences were: GLP1R, forward 5’-TAATCACCGTGGCAGGAAGAG-3’, reverse 5’-CTTGAGTGTGAGTCCGGGTT-3’, and glyceraldehyde 3-phosphate dehydrogenase (GAPDH), forward 5’-TGTGTCCGTCGTGGATCTGA-3’, reverse 5’-TTGCTGTTGAAGTCGCAGGAG-3’. Gene expression was calculated as described by (36) using GAPDH as the reference gene.

### 2.7 Experiment 1. Chow and palatable food intake after EX4 IP administration during short-term intermittent exposure to CAF diet

**Figure 1:**
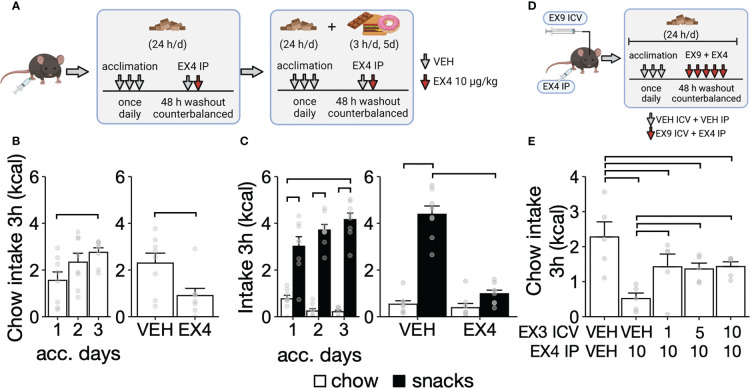
Chow and palatable food intake after EX4 IP administration during short-term intermittent exposure to CAF diet. **(A)** Experimental design for panels B-C. Mice were exposed to palatable snacks for 3h/d for 5 days, including acclimation and EX4 administration. **(B)** Chow intake during acclimation to IP injections (left) and EX4 IP administration (right, N = 8). **(C)** Chow and snacks intake during acclimation to snacks for 3 h daily (left) and after EX4 IP injection (left, N = 8). **(D)** Experimental design for panel **(E)**. **(E)** Chow intake after ICV EX3 administration followed by EX4 IP administration (N = 5). Y-axis, mean ± SEM. Brackets, P<0.05 for pairwise comparisons. Panels A and D were created with BioRender.com.

To determine whether early exposure to CAF diet altered the anorectic effects of peripheral EX4 administration, we measured food intake in response to EX4 IP administration using a within-subjects design in mice with only chow access and then short and intermittent exposure to CAF diet (**Figure 1A**). Mice with only chow access (n = 8) were acclimated to IP injections and then injected with vehicle or EX4 IP (10 μg/kg) randomized over days with 48 h between injections. Food intake was measured 3 h after each IP injection. Next, after a 10-day wash-out period without interventions, mice were acclimated to 4 palatable snacks for 3 days (3h/d; Milk Chocolate, Sugar Cookies, Cheese snacks, and Potato Chips; see **Supplementary Table 1** for commercial names and detailed nutritional information) while receiving a single daily IP saline injection. Starting on the fourth day, mice received a vehicle or EX4 IP injection (10 μg/kg) randomized over days with 48 h between injections. After IP injections, mice had access to the palatable snacks for 3 h. Chow and palatable food intake were measured 3 h post-injection. Thus, including acclimation, mice had access to the CAF diet for a total of 5 days.

To determine whether the anorectic effects of EX4 IP administration at 10 μg/kg required activation of central GLP1 receptors, we measured food intake after co-administration of IP EX4 and ICV administration of the GLP1R antagonist EX3 (**Figure 1D**). A separate group of mice (n = 5) with only access to chow were prepared with an ICV cannula and were acclimated for three consecutive days by receiving an ICV injection of aCSF 15 min. before an IP saline injection. Starting on the fourth day, mice received a vehicle or EX3 ICV injection (1, 5, and 10 ng) 15 min. before a vehicle or EX4 IP injection (10 μg/kg) with the combination of ICV and IP injections randomized over days with 48 h between the injection days. Chow intake was measured 3 hours after the EX4 IP injection.

### 2.8 Experiment 2. Chow and palatable food intake after EX4 ICV or PVN administration during short-term exposure to CAF diet

**Figure 2:**
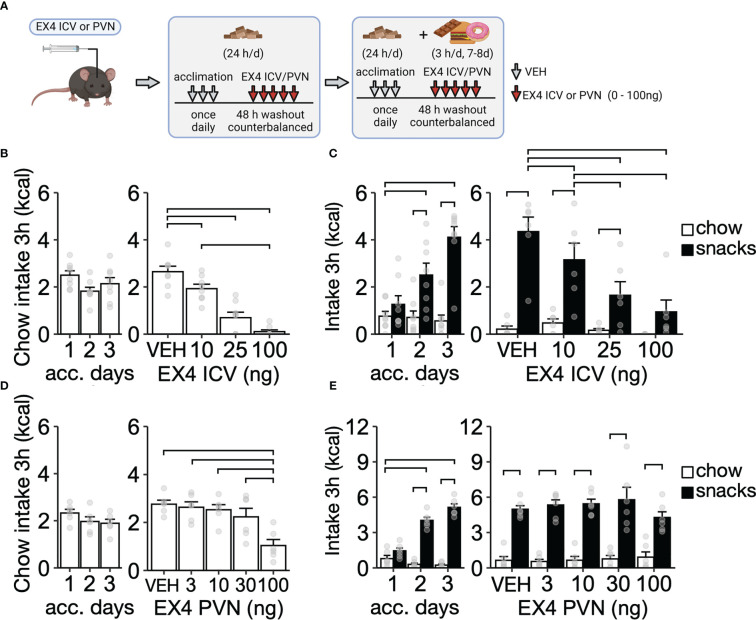
Chow and palatable food intake after EX4 ICV or PVN administration during short-term intermittent exposure to CAF diet. **(A)** Experimental design. Mice were exposed to palatable snacks for 3h/d for 7-8 days (ICV: 7 days, PVN: 8 days), including acclimation and EX4 administration. **(B)** Chow intake during acclimation to ICV injections (left) and after EX4 ICV administration (right, N = 8). **(C)** Chow and snacks intake during acclimation to snacks for 3 h/d (left) and after EX4 IP injection (right, N = 8). **(D)** Chow intake during acclimation to PVN injections (left) and after EX4 PVN administration (right, N = 8). **(E)** Chow and palatable snacks intake during acclimation to snacks for 3 h/d (left) and after EX4 PVN injection (right, N = 6). Y-axis, mean ± SEM. Brackets, P<0.05 for pairwise comparisons. Panel A was created with BioRender.com.

To determine whether early exposure to CAF diet altered the anorectic effects of central EX4 administration, we measured food intake in response to EX4 ICV or PVN administration using a within-subjects design in mice with only chow access and then with short-term and intermittent exposure to a CAF diet as described in experiment 1 (**Figure 2A**). Mice prepared with ICV (n = 10) or PVN cannulae (n = 6) were acclimated to intra-cannula injections and then injected with vehicle or EX4 ICV (10, 25, 100 ng) or into the PVN (3, 10, 30, 100 ng) with vehicle and EX4 doses randomized over days with 48 h between injections. The EX4 dosage was known to reduce intake after ICV or PVN administration (19, 37). After a 10-day washout period without interventions, mice were acclimated to 4 palatable snacks as in experiment 1 (3h/d for 3 days). Starting on the fourth day, mice were injected with vehicle or EX4 ICV or intra-PVN as before. After ICV or PVN injections, mice had access to palatable foods for 3 h after injections. Chow and palatable food intake were measured 3 h post-injection. Thus, including acclimation, mice had access to the CAF diet for a total of 7-8 days. Two mice lost their ICV cannulae during acclimation to ICV injections. Thus, the final sample size was 8 mice with ICV cannulae and 6 mice with PVN cannulae.

### 2.9 Experiment 3. Body weight, food intake, and WAT metabolism and protein expression after repeated EX4 ICV and IP administration during continuous exposure to CAF diet before induction of obesity

**Figure 3:**
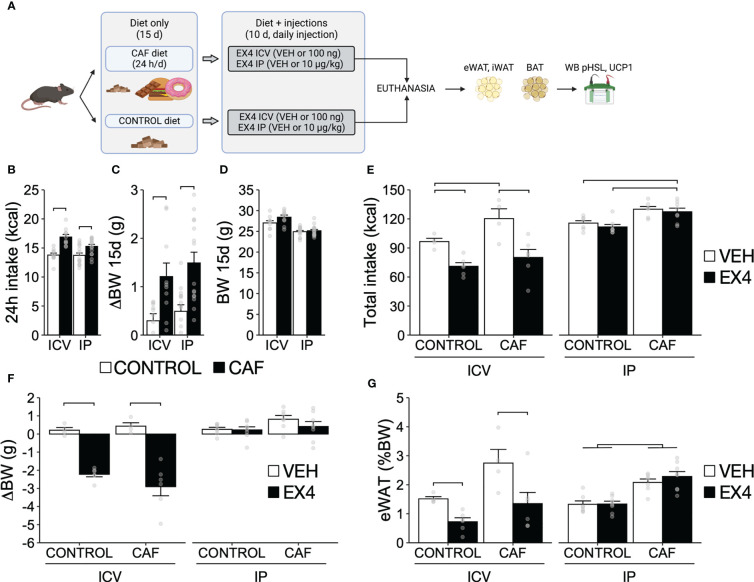
Body weight and food intake after repeated EX4 ICV and IP administration during continuous exposure to CAF diet before induction of obesity. **(A)** Experimental design. Mice fed CAF diet (N = 25) were exposed continuously (24 h/d) to palatable snacks for 15 days or remained with only control diet (chow, N = 25) before daily EX4 administration for 10d. **(B)** Daily 24 h intake (kcal), **(C)** change in body weight (∆BW), and **(D)** body weight (BW) on day 15 for mice fed CAF or control diet for 15 days before ICV and IP injection. **(E)** Total calorie intake, **(F)** ∆BW, and **(G)** Percent of epididymal WAT to final BW after 10 days of EX4 ICV (100 ng) or IP (10 µg/kg). N = 4-8 for each experimental group. Y-axis, mean ± SEM. Brackets, P<0.05 for pairwise comparisons. Panel A was created using BioRender.com.

**Figure 4:**
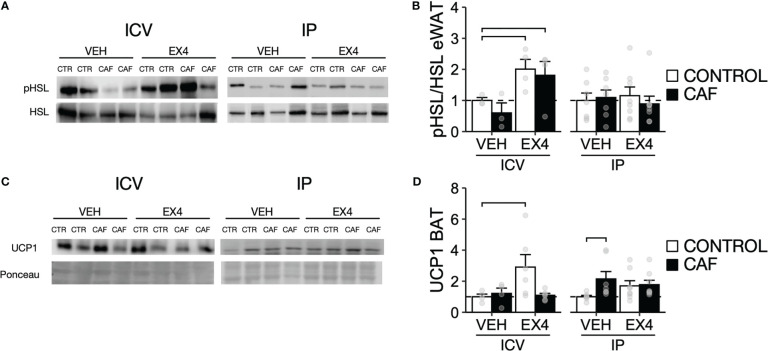
Expression of pHSL and UCP1 after repeated EX4 ICV and IP administration during continuous exposure to CAF diet before induction of obesity. **(A)** Representative images of a western blot and **(B)** quantification of optical densities of phosphorylated hormone-sensitive lipase (pHSL) and HSL in epididymal WAT (eWAT) samples from mice fed CAF diet or chow only for 15 days followed by 10d of EX4 ICV or IP administration. **(C)** Representative images of a western blot and **(D)** quantification of optical densities of UCP1 in interscapular brown adipose tissue (BAT) in mice fed CAF diet or chow only. N = 4-8 for each experimental group. Y-axis, mean ± SEM. Brackets, P<0.05 for pairwise comparisons.

To determine whether longer and continuous exposure to CAF diet before manifesting obesity reduced the anorectic effects of EX4 ICV or IP administration, we measured food intake after ICV or IP EX4 injections in mice exposed continuously to CAF or control diet (i.e., 24 h/d chow access) for 15 days (**Figure 3A**). Mice prepared with (n = 20) or without (n = 30) an ICV cannulae were singly housed and randomly assigned to CAF diet (ICV: n = 10, no surgery: n = 15) or control diet (ICV: n = 10, no surgery: n = 15) for 15 days. The CAF diet consisted of continuous access to 4 palatable snacks made for human intake that were randomly selected from 20 snacks (**Supplementary Table 1**) in addition to chow. The palatable snacks were changed every Monday, Wednesday, and Friday. After 15 days of the dietary intervention, mice were randomly assigned within each diet to receive either a daily EX4 ICV (100 ng, n = 12) or IP (10 μg/kg, n = 16) injection or their respective vehicles (aCSF for ICV, n = 8; saline for IP injections, n = 14) for 10 days while maintaining their respective diets. On the tenth day, mice were euthanized two hours after the last injection, and samples from the iWAT, eWAT, and BAT were collected to measure pHSL and UCP1 protein levels (**Figure 4**). The final group sample for mice with ICV injections was 4-6 mice per group, and for IP-injected mice was 7-8 mice per group. For mice with ICV cannulae, brains were processed to determine cannula placement. No mice were eliminated due to misplaced cannulae.

### 2.10 Experiment 4. WAT lipolysis after EX4 *ex-vivo* in mice fed CAF or control diet for 12 weeks

**Figure 5:**
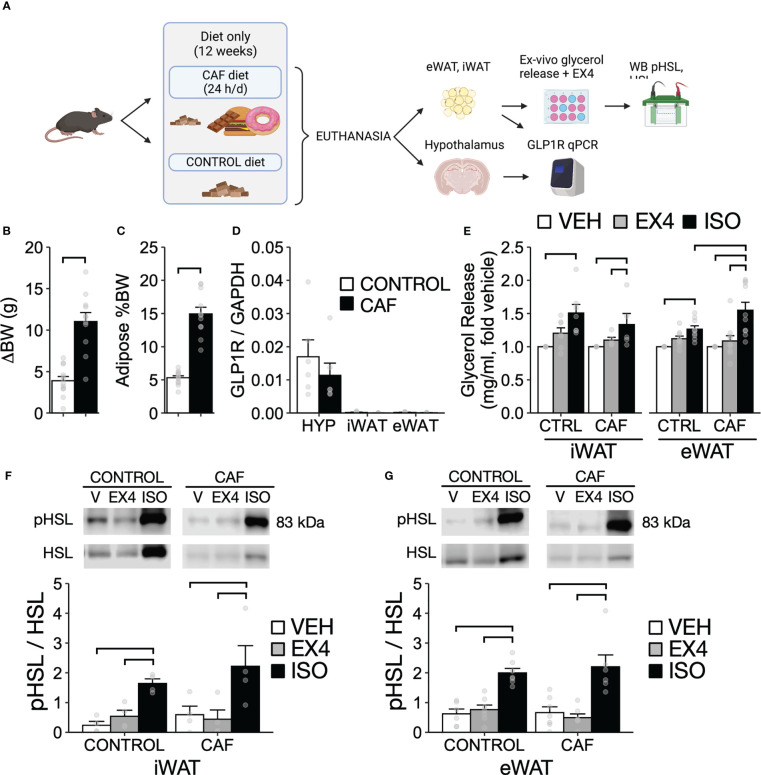
WAT lipolysis after EX4 *ex-vivo* in mice fed CAF or control diet for 12 weeks continuously. **(A)** Experimental design. **(B)** Change in body weight (BW) after 12 weeks of CAF (N = 11) or control diet (chow only, N = 12). **(C)** Percent of white adipose tissue (inguinal, mesenteric, subcutaneous, and epididymal) relative to end-point BW. **(D)** Expression of GLP1R in the hypothalamus (HYP), epididymal WAT (eWAT), and inguinal WAT (iWAT) normalized to GAPDH by ΔΔCT method (N= 6-7 per group). **(E)** Release of glycerol in WAT explants from mice fed CAF diet or chow (N = 12 for chow and N = 11 for CAF). Expression of pHSL relative to HSL in **(F)** iWAT and **(G)** eWAT (N = 4-7 per group). Y-axis, mean ± SEM. Brackets, P<0.05 for pairwise comparisons. Panel A was created with BioRender.com.

To determine whether WAT lipolysis caused by EX4 administration was mediated by GLP1R expressed in WAT and modulated by CAF diet, we examined *ex-vivo* lipolysis caused by EX4 in WAT tissue explants from mice fed CAF or control diet to EX4 (**Figure 5A**). Mice (n = 26) were group-housed and fed CAF or control diet for 12 weeks (n = 13 per diet) using the CAF diet described in experiment 3. After completing the dietary intervention, mice were euthanized during the first half of the light cycle (07:00–12:00) with excess isoflurane and the mediobasal hypothalamus, iWAT, eWAT, and mesenteric fat depots were collected. Samples from the hypothalamus were stored in RNA later^®^ (#AM7021M, Thermo Fischer), and WAT depots were snap frozen, stored at -80 °C degrees, and then used for qPCR analysis of GLP1R. Epididymal and iWAT samples were also tested for *ex-vivo* effects of EX4 on lipolysis and expression of pHSL by Western Blot. The final sample size was n = 11 and n = 12 for the CAF and control diet, respectively. One mouse per diet was removed from the study based on veterinary advice due to injuries, and one mouse fed CAF diet was excluded from the analysis because we failed to collect inguinal WAT.

### 2.11 Statistical analysis

Statistical analyses were performed using R v4.1.2. All data are presented as mean and SEM. Statistical significance was set at P < 0.05. Normality for data was examined by reviewing residual plots. For experiment 1, chow and snack intake (expressed as calories) were analyzed as separate endpoints with repeated measures ANOVA with the dose of EX4 IP as the independent variable or with the combination of EX3 ICV and EX4 IP with mice as the experimental subject. For experiment 2, chow and snack intake (expressed as calories) were analyzed as separate endpoints with repeated measures ANOVA with the dose of EX4 ICV or intra-PVN as independent variables and mice as the experimental subject. For experiment 3, the effects of the CAF diet on change in body weight, percent adiposity, and expression of GLP1R were analyzed with unpaired Student’s t-tests. Changes in glycerol release were analyzed separately for each dietary intervention (CAF vs. control) and WAT depot (inguinal and epididymal) with a repeated measures ANOVA with treatment (vehicle, EX4, and isoproterenol) as the independent variable and mice as the experimental subject. For experiment 4, daily intake and change in body weight during CAF diet feeding were analyzed with a two-way ANOVA with diet (CAF vs. control) and route of administration (IP and ICV) as independent variables. Intake, change in body weight, and percent of eWAT relative to body weight were analyzed separately with a three-way ANOVA with the interaction between dietary treatment (CAF vs. control), route of administration (ICV and IP), and EX4 dose (vehicle vs. dose) as independent variables. Changes in protein expression were analyzed separately for each protein of interest and route of administration with a two-way ANOVA with dietary intervention (CAF vs. control) and EX4 dose as independent variables. For all analyses, pairwise comparisons were done with estimated marginal means and adjusted with false discovery rate.

## 3 Results

### 3.1 Experiment 1. EX4 IP administration reduced palatable food intake during short-term exposure to a CAF diet

After acclimation to IP injections (**Figure 1B** left), EX4 IP administration reduced chow intake by 60% compared to vehicle (P < 0.05, **Figure 1B** right). During acclimation to the CAF diet (3 h/d), mice progressively ate more palatable snacks compared to chow (**Figure 1C** left; interaction between diet and days, F_2,35 = _7.23, P < 0.01). After acclimation, EX4 IP reduced intake of snacks by 75% compared to vehicle (t_21 = _10.64, P < 0.01) without altering chow intake (P = 0.64, **Figure 1C** right). Body weight did not change during the experiment (t_14 = _1.58, P = 0.13). In a separate set of mice, the reduction of chow intake by EX4 IP administration was decreased by approximately 50% by an ICV pre-treatment with EX3 (**Figure 1E**). Together, these data indicate that EX4 IP administration can reduce short-term (3 h) intake of chow and palatable snacks, an effect mediated by peripheral and central GLP1R receptors.

### 3.2 Experiment 2. EX4 ICV administration, but not intra-PVN, reduced palatable food intake during short-term exposure to CAF diet

After acclimation to ICV injections (**Figure 2B** left), EX4 ICV administration dose-dependently reduced chow intake (**Figure 2B** right; F_3,21 = _10.62, P < 0.01). During acclimation to the CAF diet (3 h/d for 3 days), mice progressively ate more palatable snacks compared to chow (**Figure 2C** left; interaction between diet and days: F_2,35 = _8.69, P < 0.01). After acclimation to palatable snacks, EX4 ICV dose-dependently reduced intake of palatable snacks (**Figure 2C** right; interaction between diet and EX4: F_3,35 = _5.06, P < 0.01). In a separate set of mice, after acclimation to PVN injections (**Figure 2D** left), EX4 PVN administration reduced chow intake (F_4,20 = _12.29, P < 0.01), an effect driven by the highest dose of EX4 tested (**Figure 2D** right). During acclimation to a CAF diet (3 h/d), mice progressively ate more snacks (**Figure 2E** left; interaction between diet and days: F_2,25 = _45.11, P < 0.01). After acclimation, EX4 PVN administration failed to reduce intake of palatable snacks (**Figure 2E**; interaction between diet and EX4: F_4,45 = _0.95, P = 0.44). Body weight did not change during EX4 ICV (t_28 = _0.44, P = 0.66) or PVN administration (t_10 = _0.56, P = 0.58). Together, these data show that the EX4 ICV or PVN administration can reduce chow intake, but only EX4 ICV administration reduces the intake of palatable snacks.

### 3.3 Experiment 3. Repeated EX4 ICV, but not IP administration, reduces food intake, causes body weight loss, and alters expression and regulation of thermogenic proteins in WAT and BAT during long-term and continuous exposure to a CAF diet before the onset of obesity

In mice prepared to receive EX4 ICV or IP administration, exposure to a CAF diet for 15 days increased daily calorie intake (**Figure 3B**; ICV: t_18 = _5.39, P = 0.01; IP: t_28 = _3.79, P = 0.01) and caused a body weight gain of ~ 1 gram (**Figure 3C**; ICV: t_18 = _2.78, P = 0.01; IP: t_28 = _3.79, P = 0.01) compared to mice fed control diet. Still, diet did not affect the final body weight on day 15 (**Figure 3D**; ICV: t_18 = _1.82, P = 0.09; IP: t_28 = _0.49, P = 0.62). After 10 days of single daily EX4 ICV or IP injections, while mice maintained their respective diets, only EX4 ICV administration reduced total intake regardless of diet (**Figure 3E**; EX4: F_1,16 = _21.69, P < 0.01; interaction between diet and EX4: F_1,16 = _1.05, P = 0.32). The same effects were observed for body weight gain and eWAT mass, as only EX4 ICV administration caused weight loss (**Figure 3F**; main effect of EX4: F_1,16 = _72.18, P < 0.01; interaction between diet and EX4: F_1,16 = _1.74, P = 0.29) and reduced eWAT (**Figure 3G**; main effect of EX4: F_1,16 = _11.86, P < 0.01; interaction between diet and EX4: F_1,16 = _0.91, P = 0.35). EX4 IP did not affect weight gain or eWAT mass (**Figures 3F, G**; P > 0.05 for all effects). These data indicate that repeated EX4 ICV, but not IP administration, can reduce intake, body weight, and adiposity during continuous access to a CAF diet.

We next examined whether the route of EX4 administration (ICV vs. IP) altered the expression of proteins related to lipolysis and thermogenesis in WAT and BAT. We selected HSL, a key enzyme in triglyceride hydrolysis that is activated by phosphorylation (38), and UCP1, an essential protein for thermogenesis (39). EX4 ICV administration increased the pHSL/HSL protein levels in mice fed with either CAF or the control diet, while EX4 IP had no effect regardless of dietary intervention (**Figures 4A, B**). Yet, in mice with EX4 ICV or IP administration, we failed to find differences in the plasma-free fatty acids independent of diet (**Supplementary Figure 1**). However, EX4 ICV administration reduced plasma triglycerides and glucose in mice fed CAF diet (**Supplementary Figure 1C–D**). EX4 ICV, but not IP administration, also increased UCP1 in BAT in mice fed control but not CAF diet (**Figures 4C, D**). However, for mice who received vehicle IP injections, UCP1 BAT levels were higher in mice fed CAF diet compared to those fed chow. Together, these data indicate that EX4 ICV administration increases pHSL in eWAT of mice fed control or CAF diet and that EX4 ICV administration increases UCP1 in BAT in mice fed control but not CAF diet.

### 3.4 Experiment 4. EX4 *ex-vivo* does not induce lipolysis in WAT explants of mice fed control or CAF diet

Mice fed a CAF diet for 12 weeks had a significantly larger body weight gain (∆BW) and percent adiposity compared to mice fed control diet (**Figures 5B, C**; ∆BW: t_21 = _6.17, P < 0.01; %adiposity: t_21 = _9.52, P < 0.01). Expression of GLP1R in the hypothalamus was significantly greater by 10-fold compared to either eWAT or iWAT depots (**Figure 5D**; F_2,21 = _21.11, P < 0.01) and GLP1R mRNA was not affected by diet (**Figure 5E**; F_1,10 = _0.89, P = 0.37). In an *ex-vivo* assay for lipolysis with isoproterenol (ISO) as a positive control for lipolytic activation, EX4 failed to increase glycerol release in eWAT and iWAT explants from mice fed CAF or control diet despite that the CAF diet enhanced the lipolytic response to isoproterenol in eWAT (**Figure 5E**). EX4 *ex-vivo* also failed to increase pHSL expression in the explants from both WAT depots independent of diet. In contrast, ISO increased pHSL in all WAT depots without difference between diets (**Figures 5F, G**). Overall, consistently with low levels of GLP1R expression in eWAT and iWAT regardless of diet, direct application of EX4 failed to increase lipolysis in eWAT and iWAT from mice fed either CAF or control diet.

## 4 Discussion

This study aimed to understand how exposure to an obesogenic food environment could alter the effects of EX4 on food intake, weight loss, and adipose tissue metabolism. We first tested whether different schedules of CAF diet before the onset of obesity could reduce the anorectic, lipolytic, and thermogenic effects of GLP1R activation by peripheral and central EX4 administration. Second, we tested whether *ex-vivo* EX4 would increase WAT lipolysis and if long-term exposure to a CAF diet influenced lipolysis. Our data show that, while EX4 ICV and IP administration can reduce intake of palatable snacks during short-term exposure to a CAF diet (3 h/d for five to eight days), only EX4 ICV can reduce intake and body weight in mice with continuous (24 h/d for 15 days) access to a CAF diet. Finally, *ex-vivo* EX4 does not increase lipolysis in eWAT and iWAT explants from lean mice fed a control diet or obese mice fed a CAF diet for 13 weeks.

We show that EX4 ICV or IP administration, but into PVN, can reduce palatable food intake during intermittent short-term access to a CAF diet (**Figures 1B**, **2B**). However, EX4 reduced chow intake regardless of the administration route. The anorectic effect of EX4 on chow was reduced by a previous ICV administration of the GLP1R antagonist EX3 (**Figure 1C**). However, as EX3 was injected into the ventral third ventricle, its diffusion most likely only reached periventricular hypothalamic nuclei (40, 41), while the anorectic actions of EX4 involve vagal-mediated effects and central effects that engage hypothalamic and extra-hypothalamic GLP1R (15, 42). Thus, it is unlikely that EX3 administration could have blocked all central effects of EX4 in this study.

The effects of EX4 in PVN on chow intake were smaller relative to both EX4 ICV and IP, which is consistent with the expected engagement of more brain sites that express GLP1R by ICV or IP EX4 administration. We previously showed that EX4 IP in Balb/c male mice reduced chow intake but could not block palatable food intake during short-term access (3 h/d for seven days) to a CAF diet (21). In the studies presented here, we used male C57BL6/J mice; thus, strain differences might account for the differences in sensitivity to EX4 IP administration. The lack of effects of EX4 into the PVN on intake of CAF diet was unexpected, as PVN receives strong innervation of PPG-NTS neurons and activation of the GLP1R receptors in this brain region can inhibit chow intake (16–18). Because ICV injection into the ventral 3^rd^ ventricle would be expected to engage more brain sites than just PVN, this result suggests that the activation of the GLP1R over several brain sites is necessary to reduce palatable food intake.

Although EX4 ICV or IP administration can reduce the intake of chow and palatable snacks during short-term intermittent access to a CAF diet, only repeated administration of the same ICV EX4 dose reduced intake during continuous and longer exposure to CAF diet for 15 days. We previously showed that exposing mice to a CAF diet for ten weeks caused obesity and blocked the anorexigenic effects of EX4 IP administration on the intake of palatable snacks (21). In this study, mice only had access to a CAF diet for two weeks, which caused hedonic intake (indicated by increased intake of palatable food relative to chow) and a small (~1 g) weight gain (**Figure 3C**). Still, this exposure to the CAF diet failed to induce absolute differences in body weight (**Figure 3D**), which suggests that mice were in the early stages of obesity development compared to mice fed a CAF diet for 12 weeks (**Figure 5**). Although a higher dose of EX4 IP in mice with continuous access to a CAF diet might have reduced intake and caused body weight loss, we highlight the contrasting results between the effects of EX4 IP during short-term exposure to CAF (3 h daily every other day for a total of 5 days including acclimation) and longer exposure to CAF (continuous access for a total of 25 days including acclimation). This result supports that chronic intake of palatable foods can alter the response to EX4 during the early stages of obesity, which might contribute to further increases in food intake and body weight over time.

It is possible that the extended period of the CAF diet could have also reduced the transport of EX4 into the central nervous system, which depends on passive mechanisms (43) as well as active endothelial transport dependent on GLP1 receptor activity (44, 45). However, whether tanycytes mediate EX4 transport into the brain has not been examined (46). Future studies should assess whether CAF diet feeding reduces EX4 transport across the blood-brain barrier.

Consistent with the differences in their effects on adiposity, EX4 ICV but not IP administration increased pHSL expression regardless of diet, and only EX4 ICV in mice fed chow increased BAT UCP1 expression (**Figure 4**). The latter result is consistent with data showing that activation of the GLP1R in the dorsal medial hypothalamus (a brain site likely reached by EX4 ICV administration into the third ventricle) can increase BAT thermogenesis (47). Our data show that CAF diet exposure blocked this effect, suggesting that the weight loss caused by EX4 ICV is primarily due to a reduction in intake rather than the thermogenesis mediated by UCP1 in BAT. However, confirming this hypothesis would require direct measurements of energy expenditure. In mice fed a CAF or control diet for 15d, EX4 ICV reduced plasma TG and glycemia. However, EX4 ICV failed to reduce plasma FFA regardless of diet. While in mice fed the control diet, this could suggest higher clearance of FFA from plasma due to increased UCP1 expression in BAT (25, 27, 28, 31), this effect is absent in mice fed CAF diet. Further examination of the effects of EX4 and CAF diet on WAT metabolism would be needed to support this hypothesis. Still, these effects are observed without the manifestation of obesity, suggesting they might reflect a metabolic dysfunction in WAT associated with inflammation caused by poor dietary quality (22, 23, 48) as mice fed CAF diet continued to consume more calories from palatable snacks even during EX4 injection.

We demonstrated that EX4 failed to increase lipolysis in eWAT or iWAT explants *ex-vivo*. This is consistent with a lack of effects in pHSL and the low levels of GLP1R mRNA detected in these tissues compared to those in the hypothalamus. These data align with data from transgenic mice lacking GLP1R WAT expression (49) and the hypothesis that the effects of activating GLP1R on WAT metabolism are mediated by activation of the sympathetic nervous system (33). The lack of effect of EX4 on pHSL expression in eWAT or iWAT *ex-vivo* (**Figure 5**) is also consistent with the lack of effects of repeated EX4 IP on pHSL in either tissue, but that EX4 ICV increased pHSL in both WAT depots regardless of diet. Together, these data support the conclusion that eWAT and iWAT do not express a functional GLP1R (33).

Our data have clinical implications. Our data support that exposure to the obesogenic environment, without the development or manifestation of obesity, is sufficient to alter the response to GLP1R agonists. Further, these data suggest that the anorectic effects of GLP1R activation also depend on the duration of the exposure to the obesogenic environment. Together, these data highlight the need to consider the individual food environment and personal history in the success of anti-obesity interventions for GLP1R agonists. Our data also suggest that central administration of GLP1R agonists is more effective in causing weight loss than peripheral administration, which might impact the pharmacological designing of new GLP1R agonists over the following years to favor their brain actions. Similarly, this study, and future ones, will help to understand which tissues expressing GLP1R are essential in the anorectic effects of GLP1R agonists. Overall, our findings have future implications in therapeutical modifications of current GLP1 agonists, considering exposure, administration route, and tissue specificity.

In conclusion, we showed that either ICV or peripheral EX4 administration reduced palatable food intake during intermittent and short exposure to a CAF diet. However, EX4 administration into PVN failed to reduce CAF diet intake. Yet, during continuous and longer exposure to a CAF diet over 15 days before the development of obesity, only central activation of GLP1R reduced intake and caused weight loss. Yet, the same duration of exposure to a CAF blocked the increase in UCP1 in BAT caused by EX4 ICV administration. Finally, we demonstrated that WAT expression of GLP1R and activation are marginal in mice regardless of whether the animals are fed control or CAF diet. Overall, these results support the concept that exposure to the obesogenic food environment without or before the development or manifestation of obesity can alter the response to GLP1R agonists and that the effects of GLP1R activation on WAT are not mediated by the direct action of GLP1R agonists on this tissue regardless of obesity.

## Data availability statement

The raw data supporting the conclusions of this article will be made available by the authors, without undue reservation.

## Ethics statement

The animal study was reviewed and approved by Institutional Animal Care and Use Committee at Pontificia Universidad Catolica de Chile.

## Author contributions

CP-L and PM conceived and designed the research; CP-L, PM, and CJ performed the experiments. CP-L, PM, and CJ analyzed data and prepared the figures. CP-L drafted the manuscript. All authors contributed to interpreting experiments, editing, and revising the manuscript. All authors contributed to the article and approved the submitted version.
