# Corrigendum: Impact of short and long exposure to cafeteria diet on food intake and white adipose tissue lipolysis mediated by glucagon-like peptide 1 receptor

**DOI:** 10.3389/fendo.2023.1240246

**Published:** 2023-06-26

**Authors:** Pamela Mattar, Cristian Jaque, Jennifer A. Teske, Eugenia Morselli, Bredford Kerr, Víctor Cortés, Rene Baudrand, Claudio E. Perez-Leighton

**Affiliations:** ^1^ Facultad de Ciencias Biológicas, Pontificia Universidad Católica de Chile, Santiago, Chile; ^2^ Department of Physiology, School of Nutritional Sciences and Wellness, Graduate Interdisciplinary Programs in Physiological Sciences and Neuroscience, University of Arizona, Tucson, AZ, United States; ^3^ Department of Food Science and Nutrition at the University of Minnesota, Saint Paul, MN, United States; ^4^ Department of Basic Sciences, Faculty of Medicine and Sciences, Universidad San Sebastián, Santiago, Chile; ^5^ Centro de Biología Celular y Biomedicina-CEBICEM, Facultad de Medicina y Ciencia, Universidad San Sebastián, Santiago, Chile; ^6^ Department of Nutrition, Diabetes, and Metabolism, Faculty of Medicine, Pontificia Universidad Católica de Chile, Santiago, Chile; ^7^ Department of Endocrinology, Faculty of Medicine, Pontificia Universidad Catolica de Chile, Santiago, Chile; ^8^ Centro Traslacional de Endocrinologia UC CETREN, Pontificia Universidad Catolica de Chile, Santiago, Chile

**Keywords:** obesity, glucagon-like peptide 1 (GLP-1), cafeteria diet, lipolysis, white adipose tissue

## Incorrect Affiliation

In the published article, there was an error in affiliation 8. Instead of “Department of Urology, Faculty of Medicine, Pontificia Universidad Catolica de Chile, Santiago, Chile”, it should be “Centro Traslacional de Endocrinologia UC CETREN, Pontificia Universidad Catolica de Chile, Santiago, Chile”.

The authors apologize for this error and state that this does not change the scientific conclusions of the article in any way. The original article has been updated.

