# Supplementary material for: Impact of short and long exposure to cafeteria diet on food intake and white adipose tissue lipolysis mediated by glucagon-like peptide 1 receptor
Source: Front Endocrinol (Lausanne). 2023 May 24;14:1164047. doi: 10.3389/fendo.2023.1164047 (PMC10244886; doi:10.3389/fendo.2023.1164047)
Supplement: Supplementary file 1 [file DataSheet_1.docx]

Supplementary Material

Impact of short and long exposure to cafeteria diet on food intake and white adipose tissue lipolysis mediated by glucagon-like peptide 1 receptor.

**Mattar P1, Jaque C1, Teske JA2,3, Morselli EM4, Kerr B5, Cortes VC6, Baudrand R7,8, Perez-Leighton CE1*.**

*** Correspondence:**Claudio Perez-Leighton.

Email: cperezl@bio.puc.cl.


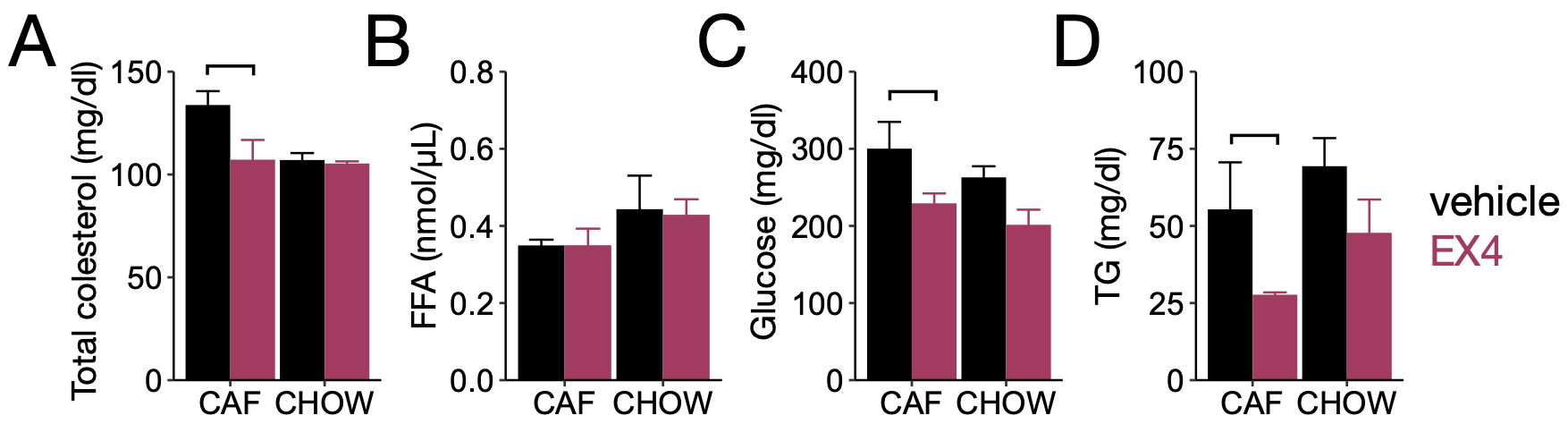


**Supplementary Figure 1. Effect of repeated EX4 ICV on serum parameters.** Y-axis, mean ± SEM. Brackets, P<0.05 for pairwise comparisons.

**SUPPLEMENTARY TABLE 1. Description of cafeteria diet snacks**

| **Description** | **Commercial Name** | **Brand** | **Kcal / 100 g** | **Proteins / 100g** | **Total fat / 100g** | **Total carbohydrates / 100g** | **Total sugars / 100 g** | **Sodium (mg)** |
| --- | --- | --- | --- | --- | --- | --- | --- | --- |
| Chocolate chip cookies | Toddy | Pepsico | 479 | 5.6 | 21 | 68 | 34 | 461 |
| Cookie filled with peanut butter flavored cream and covered in chocolate | Bon-o-bon | Arcor | 533 | 8.3 | 30 | 57 | 41 | 104 |
| Chocolate flavored cookie | Galletas de Chocolate | Costa | 481 | 4.1 | 21.3 | 68.2 | 28.7 | 363 |
| Milk chocolate | Trencito | Nestlé | 548 | 8.3 | 30.5 | 60 | 52.3 | 108 |
| Butter flavored cookies | Galletas de mantequilla | Costa | 479 | 4 | 20.2 | 70.5 | 28.7 | 312 |
| Wanilla wafers sprinkled with sugar | Criollitas | Costa | 400 | 7.4 | 5.4 | 80.1 | 54 | 109 |
| Oat breakfast cereal | Cuadraditos avena | Quaker | 361 | 11 | 4.9 | 68 | 12 | 339 |
| Fruit flavored breaksfast cereal | Cereal desayuno Trix | Nestlé | 380 | 4.7 | 5.3 | 78.3 | 29.9 | 280 |
| Peanut nougat | Mantecol | Mondelez | 503 | 14 | 31 | 42 | 40 | 11 |
| White chocolate | Chocolate blanco | Mondelez | 532 | 4.3 | 28 | 64 | 64 | 150 |
| Fried cheese flavored snacks | Cheetos | Evercrisp | 464 | 6.6 | 18 | 59 | 2.8 | 390 |
| Ham flavored crackers | Twistos Sabor Jamon | PepsiCo | 478 | 12 | 18 | 66 | 4 | 497 |
| Doritos Sabor Queso | Doritos Sabor Queso | Evercrisp | 490 | 5.9 | 26 | 59 | 2.3 | 495 |
| Cereal horneado sabor maní, Crunchis | Cereal horneado sabor maní, Crunchis | Marco polo | 476 | 8.7 | 21.9 | 60.9 | 1.8 | 551 |
| Potato chip flavor snack | Ramitas | Evercrsp | 533 | 8.4 | 26 | 60 | 1.2 | 452 |
| Salted fried crackers | Galletas cracker | Selz | 450 | 11 | 17 | 64 | 0.8 | 672 |
| Salted potato chips | Lays | Evercrisp | 530 | 3.6 | 32 | 57 | 3.3 | 500 |
| Butter flavored popcorn | Cabritas de mantequilla | Marco Polo | 492 | 8.1 | 26 | 56 | 6.2 | 546 |
| Unsalted almonds | Almendras | Marco Polo | 567 | 21.2 | 49.4 | 9.5 | 3.9 | 1 |
| Unsalted peanuts | Peanut | Marco Polo | 588 | 25.6 | 49.2 | 10.7 | 4.6 | 39.3 |
